# Oscillatory Neural Correlates of Police Firearms Decision-Making in Virtual Reality

**DOI:** 10.1523/ENEURO.0112-24.2024

**Published:** 2024-07-26

**Authors:** Nicholas A. Alexander, Clíona L. Kelly, Hongfang Wang, Robert A. Nash, Shaun Beebe, Matthew J. Brookes, Klaus Kessler

**Affiliations:** ^1^Wellcome Centre for Human Neuroimaging, Department of Imaging Neuroscience, UCL Queen Square Institute of Neurology, University College London, London WC1N 3AR, United Kingdom; ^2^Aston Institute of Health and Neurodevelopment, College of Health and Life Sciences, Aston University, Birmingham B4 7E, United Kingdom; ^3^Yale Child Study Center, Yale University School of Medicine, New Haven, Connecticut 06520; ^4^School of Psychology, University College Dublin, Dublin D4, Ireland; ^5^Sir Peter Mansfield Imaging Centre, School of Physics and Astronomy, University of Nottingham, Nottingham NG7 2QX, United Kingdom

**Keywords:** decision-making, expertise, naturalistic stimuli, neural oscillations, police, virtual reality

## Abstract

We investigated the neural signatures of expert decision-making in the context of police training in a virtual reality–based shoot/don’t shoot scenario. Police officers can use stopping force against a perpetrator, which may require using a firearm and each decision made by an officer to discharge their firearm or not has substantial implications. Therefore, it is important to understand the cognitive and underlying neurophysiological processes that lead to such a decision. We used virtual reality–based simulations to elicit ecologically valid behavior from authorized firearms officers (AFOs) in the UK and matched novices in a shoot/don't shoot task and recorded electroencephalography concurrently. We found that AFOs had consistently faster response times than novices, suggesting our task was sensitive to their expertise. To investigate differences in decision-making processes under varying levels of threat and expertise, we analyzed electrophysiological signals originating from the anterior cingulate cortex. In line with similar response inhibition tasks, we found greater increases in preresponse theta power when participants inhibited the response to shoot when under no threat as compared with shooting. Most importantly, we showed that when preparing against threat, theta power increase was greater for experts than novices, suggesting that differences in performance between experts and novices are due to their greater orientation toward threat. Additionally, shorter beta rebounds suggest that experts were “ready for action” sooner. More generally, we demonstrate that the investigation of expert decision-making should incorporate naturalistic stimuli and an appropriate control group to enhance validity.

## Significance Statement

This study aims to unravel the complexities of how expertise affects neural processes during uncertain scenarios by investigating police decision-making. We present our variant on shoot/don't shoot tasks, which was codeveloped with police instructors to allow graded levels of force to elicit realistic responses. We show that experts exhibit superior performance in this virtual reality–based task and that this is associated with greater modulation of frontal midline theta activity prior to a decision. Understanding the intricacies of police decision-making—especially concerning the use of firearms—is vital to inform policy effectively. Further, the naturalistic imaging methods employed here hold a broader significance for neuroscientists aiming to investigate real-world behavior.

## Introduction

Authorized firearms officers (AFOs) of the UK police forces can be authorized to discharge a firearm within the bounds of domestic and international law (Secretariat, United Nations, 1990). While their intention is to apply stopping force, the result may be lethal. To inform policy, deployment, and training aiming to minimize harm, it is imperative that effort is made to understand the cognitive and underlying neurophysiological processes related to police decision-making and expertise (Rogers, 2003). We based our predictions on prior lab-based research on neural signatures of action-related decision-making (Walsh and Anderson, 2012; Cavanagh and Frank, 2014; Eisma et al., 2021) expecting that basic aspects of neural processing would generalize to realistic police-type decision-making. In turn, we expected that our findings within this crucial field of investigation would provide unique insight into how specialized training in decision-making may affect brain signatures more generally.

Recent reviews of research into police decision-making have argued that they do not consistently meet high methodological standards (Cox et al., 2014; Hope, 2016). For example, novices, rather than police officers, are often studied (Correll et al., 2006; Pleskac et al., 2018; Scott and Suss, 2019), and control groups are not always matched across demographics, such as age (Nieuwenhuys et al., 2012a; Brisinda et al., 2015; Landman et al., 2016; D. J. Johnson et al., 2018; Hamilton et al., 2019; Taylor, 2020), limiting the generalizability and validity of findings, respectively. Despite this, studies of police decision-making have benefited from the use of naturalistic stimuli to promote ecological validity of results and to replicate the stress induced by real-world firearms incidents that are not emulated in a standard computer-based task (Cox et al., 2014; Sonkusare et al., 2019), and recent shoot/don't shoot (SDS) studies have taken advantage of this method (R. R. Johnson et al., 2014; Taylor, 2020; Biggs and Pettijohn, 2022). Further, developments in virtual reality (VR) technology (Slater, 2018) provide an opportunity for even greater immersion and interactivity while still maintaining a high level of experimental control (de la Rosa and Breidt, 2018).

In the current study, we created an SDS task presented using a head-mounted display (HMD)–based VR, enabling participation with negligible prior training specific to the experiment and equipment. This allowed us to study expert AFO participants, as well as a control group of age- and sex-matched nonpolice, novice participants, while they engaged with dynamic, naturalistic scenarios in VR. Based on the expert advice of police instructors, we adapted the standard SDS task (Correll et al., 2002) by using immersive VR to present scenarios with graded threat levels and realistic decisions that were split into two phases, a threat assessment phase and a response phase. This ensured a closer link to real-world police training and conflict situations.

To study components of electroencephalography (EEG) that are of interest to SDS decision-making, we employed combined VR-EEG methods. In particular, frontal midline theta (FMθ) neural oscillations are related to action selection and initiation of executive control (Cavanagh and Frank, 2014; Eisma et al., 2021) in decision-making under uncertainty (Walsh and Anderson, 2012). While the effects of expertise on SDS decision-making are poorly understood, we can draw on studies of similar response inhibition tasks to form hypotheses about electrophysiological differences between SDS task conditions. For instance, expert athletes in open skill sports, like tennis, perform better than novices in Go/No-Go tasks and present with earlier and greater amplitude N200 event-related potential when inhibiting a response (Di Russo et al., 2006; You et al., 2018), emphasizing the importance of training and expertise as contributing factors in decision-making under uncertainty.

Our improved task, which was co-designed with police instructors, along with concurrent EEG, allowed for unprecedented insight into the decision processes of police firearms experts during assessment (Phase 1) and response (Phase 2) to threatening scenarios that significantly extended beyond previous findings from earlier SDS paradigms. We expected group differences in performance for both decision phases, with the expert group being faster at both decision-making phases. Differences in response time between conditions in SDS tasks have been consistently observed (Correll et al., 2002; Nieuwenhuys et al., 2012b), where the decision to shoot is faster than the decision not to shoot. From our analysis of EEG neural oscillations during decision-making, we expected stronger FMθ for experts than matched controls to emerge at the preparation phase. We also expected experts to elicit greater FMθ than novices in the SDS phase. However, based on previous research, the don't shoot condition of our SDS task should be associated with longer reaction times and greater FMθ than the shoot condition, an effect that could potentially be more pronounced in experts. Finally, the successful extraction of meaningful spectral signatures such as FMθ in a dynamic VR scenario would provide a crucial proof of concept for future EEG-VR studies of expert decision-making. Such studies would increase realism and therefore the validity of neurocognitive findings.

## Materials and Methods

### Participants

The experiment was completed by participants at three centers in the UK: Expert AFOs completed the study at a police training center; novice participants completed the study within a comparable physical context at either Aston University or the University of Nottingham. All participants gave their informed consent to participate in this study. The study was approved by the Aston University Research Ethics Committee.

The Expert AFO group included 27 police officers with up-to-date training (College of Policing, Police Firearms Training Curriculum). Their ages ranged from 27 to 53 (*M* = 40.6, SD = 6.8), 26 were male, and 2 were left-handed (Fig. 2*B*). Their experience as police officers ranged from 5 to 32 years (*M* = 17.1, SD = 6.9), and they had been AFOs for between 1 and 22 years (*M* = 10.6, SD = 7).

We also recruited a Matched Novice control group of novice participants. Their ages ranged from 27 to 55 years (*M* = 38.3, SD = 8.9), 26 were male, and 2 were left-handed. In addition, we collected data from an Unmatched Novice group, with demographics (age and sex) representative of a typical experiment cohort. This group was made up of 30 participants, but 3 were excluded from analysis during data collection due to experimenter error. The 27 remaining participants’ ages ranged from 18 to 25 (*M* = 20.4, SD = 2.6), 12 were male, and 3 were left-handed.

### Virtual reality setup

#### Head-mounted display

An Oculus Rift CV1 (Meta Platforms) HMD presented the experiment as a 3D virtual environment using displays with a combined field of view of 110° and 1,080 × 1,200 resolution per eye at a 90 Hz refresh rate. Participants responded using two Oculus Touch controllers held in their hands. They wore an EEG cap underneath the HMD. A speaker in the room was used for presenting audio, as the Oculus Rift CV1 headphones were not used, to reduce electrical artifacts.

**Figure 1.**
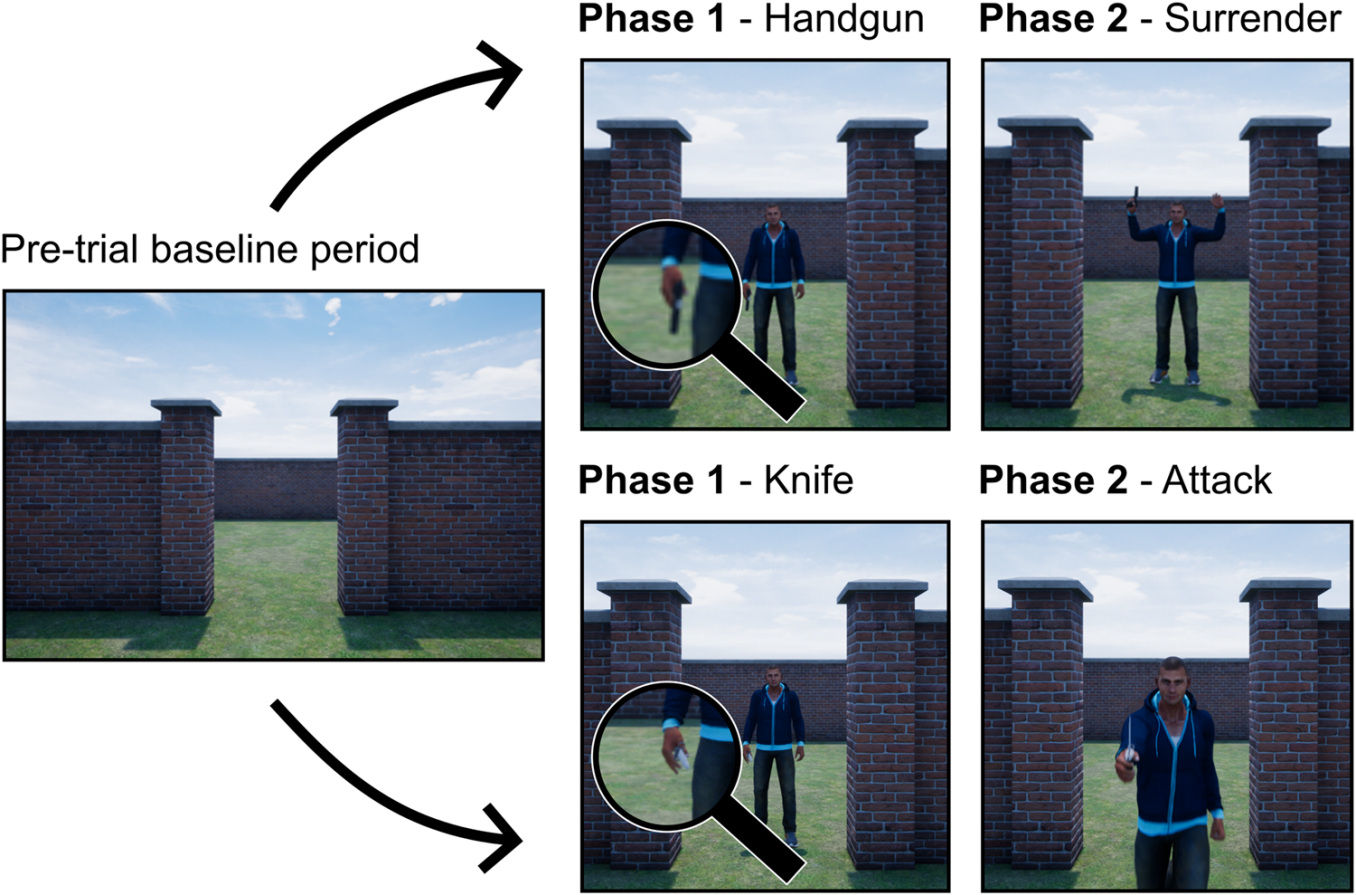
Examples of stimuli from two scenarios. Between the pretrial baseline period and Phase 1, the virtual human walked from behind the wall, into view. Two examples of threat at Phase 1 can be seen (handgun and knife), as well as the two possible outcomes at Phase 2 (surrender and attack). Note that these examples are snapshots from a continuous stimulus presentation to an HMD, so the precise presentation varied continuously with participant movement, and the display technology presented it as a high-resolution 3D scene.

The virtual human and environment were produced using Unreal Engine 4 (Epic Games). The environment comprised two walled courtyards separated by another wall with an opening in the middle, which participants faced at the start of each trial (Fig. 1). From their perspective, the virtual human started each trial in the opposite courtyard, on the right, concealed by the dividing wall. A single virtual human was used for all trials: a Caucasian male, casually dressed and with a neutral expression.

#### Action mapping

Participants used virtual hands to engage with the task. Four buttons on the hand controller allowed them to do this: a trigger for the index finger, a trigger for the middle finger, and two buttons for the thumb. The middle finger trigger was used for grabbing firearms, the index trigger for discharging firearms, and the thumb buttons for pressing safety and indicating readiness to continue. Triggers could only be used on the dominant hand controller.

Two virtual holsters held both a Glock (a self-loading pistol/handgun used by AFOs) and a Taser (a conducted energy device provided as a less lethal alternative use of force). The Glock was placed close to the hip on the dominant side. The Taser was placed at the center of the chest. Firearms instructors advised that these are common placements for AFOs. The placement was approximate as we used head tracking information only, and we assumed the chest was just below the head and the hips further below and to the side. In order to grab a firearm using the middle finger, participants first had to move their hands to the position of the desired virtual holster. The onset of this movement was not recorded, only the grabbing action itself.

### Procedure

#### General procedure

Regardless of expertise, participants were briefed in the same way and given the same instructions. The HMD was set up and adjusted for comfort and clarity of display. Participants then practiced the task under instruction until familiar. EEG was next applied, and the participant was coached through minimizing artifacts, by demonstrating the effect of talking and moving muscles on the recording. They then completed the main experiment, which was made up of 10 blocks of 20 trials, which took ∼35 min.

#### Task procedure

Based on the firearms instructor’s advice, each trial had two phases, risk assessment/preparation (Phase 1) and SDS decision (Phase 2; Fig. 1). The weapon presence condition determined the stimulus presented at the preparation stage, and virtual human compliance determined the stimulus presented at the decision stage. Participants made one response per stage in most instances. The only exception was when participants discharged their firearms but missed the virtual human on the first (and possibly subsequent) attempt. Note that, in these instances, the trial was excluded from the analysis. Participants were instructed that all responses should be made as quickly and accurately as possible.

At Phase 1, the virtual human would walk from his starting position behind the right wall (from the participant's perspective, completely obscured) to stand at the entrance in front of the participant. In their hands, they could hold a gun, a knife, or a drinks can. Previous research has typically only compared a gun to a neutral condition (Correll et al., 2006; Nieuwenhuys et al., 2012b). Our manipulation of three threat levels and a choice of stopping force was again based on the advice of firearms instructors to produce a realistic task in line with expected behavior from AFOs. The weapon presence condition (Fig. 1) determined which item was held in the virtual human's hand and would appear as he came into view. Participants then completed one of three preparation actions: equip Glock, equip Taser, or press safety. Their instructions told them that the correct preparation for each weapon presence condition was to equip the Glock if the suspect held a handgun, Taser if they held a knife, and press safety if they held a drinks can. Whichever action participants completed first was recorded as their response: They could not change their minds during a trial. When safety was pressed, a “click” sound was made, and the firearms could no longer be equipped. When a firearm (either Glock or Taser) was equipped, it could not be dropped or replaced with the other firearm. If equipped, participants were instructed to aim the firearm at the center of mass of the virtual human, in preparation for the next stage.

The decision stage began when the virtual human either attacked or surrendered, as determined by the compliance condition. The surrender animation was always the same—the virtual human would raise both hands while standing in place. The attack animation for the handgun involved the virtual human raising their hand to point it toward the participant. For the knife, the same arm animation was used, but the virtual human also moved toward the participant. All animations took 1 s to complete.

Participants could decide to either discharge their firearm or press the safety button. Whichever action they chose disabled the other. They were instructed to press safety as soon as they saw the virtual human surrendering. A click sound gave them feedback to let them know the button had been pressed. Likewise, they were to shoot as soon as the virtual human attacked. If they missed their shot, they were permitted to take another one. When the virtual human was shot, his animation blended into a new animation, and he fell to the ground. When the safety was pressed, the animation continued to its end. At the end of a trial, the virtual human disappeared, and any equipped firearms were automatically replaced in their holster.

Each of the possible combinations of Phase 1 and Phase 2 (drinks can, surrender; knife, attack; knife, surrender; handgun, attack; handgun, surrender) was repeated 40 times, balanced across the 10 blocks.

### EEG analysis

#### Online EEG recording

EEG data were recorded using an eego sports system (ANT Neuro) with 65-electrode (Ag/AgC) gel-based waveguard EEG caps, which followed the 10–10 extension of the International 10–20 system for electrode placement (Jasper, 1958). During recording, electrodes were referenced to the CPz electrode and grounded at position AFz. All electrodes were continuously sampled at 500 Hz, and the median impedance of electrodes across all recordings was 10.2 kΩ. Triggers sent via a parallel port from the computer rendering the experimental stimulus presented on the HMD were used to record stimulus presentation and behavioral responses of participants directly into the EEG time series. The data were organized into a Brain Imaging Data Structure (BIDS) compatible format (Niso et al., 2018).

#### Preprocessing

Two electrodes (M1, M2) were found to be corrupted by movement artifacts and were removed before preprocessing. EEG data were then rereferenced to the common average of all remaining electrodes. We applied Zapline (de Cheveigné, 2020) at 50 Hz (500 ms overlapping window, one component) to remove line noise and 52.1 Hz (500 ms component, three components) to remove AC noise specific to the HMD (Weber et al., 2021). Next, data were bandpass filtered [0.5–120 Hz passband, zero-phase, two-pass (forward and reverse), Hamming-windowed, fourth-order digital Butterworth filter, −24 dB/octave slope].

The continuous data were segmented into epochs defined from 3 s before to 3 s after each trial where the participant responded correctly. Each epoch was visually inspected for artifacts, and if any were present, the whole epoch was excluded. An independent components analysis (ICA) using the infomax algorithm (Bell and Sejnowski, 1995) was used to identify 61 independent components. Components identifiable as artifacts (eyeblink, eye movement, muscle activity, channel movement, electrocardiogram) were removed (mean, 5.5 components per dataset). The process of visual inspection and ICA was iterative, as artifacts that could not be successfully removed with ICA were instead removed at the trial level and vice versa. For example, if when inspecting a component, it was apparent that it was local to one trial (e.g., brief channel movement), we would remove the trial and rerun the ICA, rather than removing the whole component. Only a single ICA decomposition was performed per dataset. We did not attempt to use any automated artifact rejection techniques as our sample size was small enough for visual inspection to be possible.

#### Creating the forward model

Individual head models were created from 3D scans taken of participants while wearing the EEG cap. Following an established electrode digitization pipeline (Homölle and Oostenveld, 2019), electrodes were localized by labeling the model and moving the electrode position inwards by 8 mm (electrode thickness). When permitted by participants’ hair, head positions on the forehead and back of the head were also measured. Electrodes and head positions were then parsed as head digitization coordinates to Brainstorm (Tadel et al., 2019). Within Brainstorm, a template anatomy [ICBM152 2016c (Fonov et al., 2009)], which included a T1 structural MRI and boundary element model (BEM) of the scalp, skull, and brain (Oostendorp and van Oosterom, 1989), was warped to fit these landmarks using a nonlinear transformation (Ashburner and Friston, 1997).

The template and individual head models were used in FieldTrip for further analysis. A leadfield was created for the template using the “dipoli” method in FieldTrip with default conductivity values (Gabriel et al., 1996). For each individual, a nonlinear transformation between the individual and template head model was calculated (Friston et al., 1995). The inverse of this transformation was then applied to the template leadfield, and the output was used as the individual leadfield, allowing subsequent analysis to be conducted in a common space.

#### Source localization

**Figure 2.**
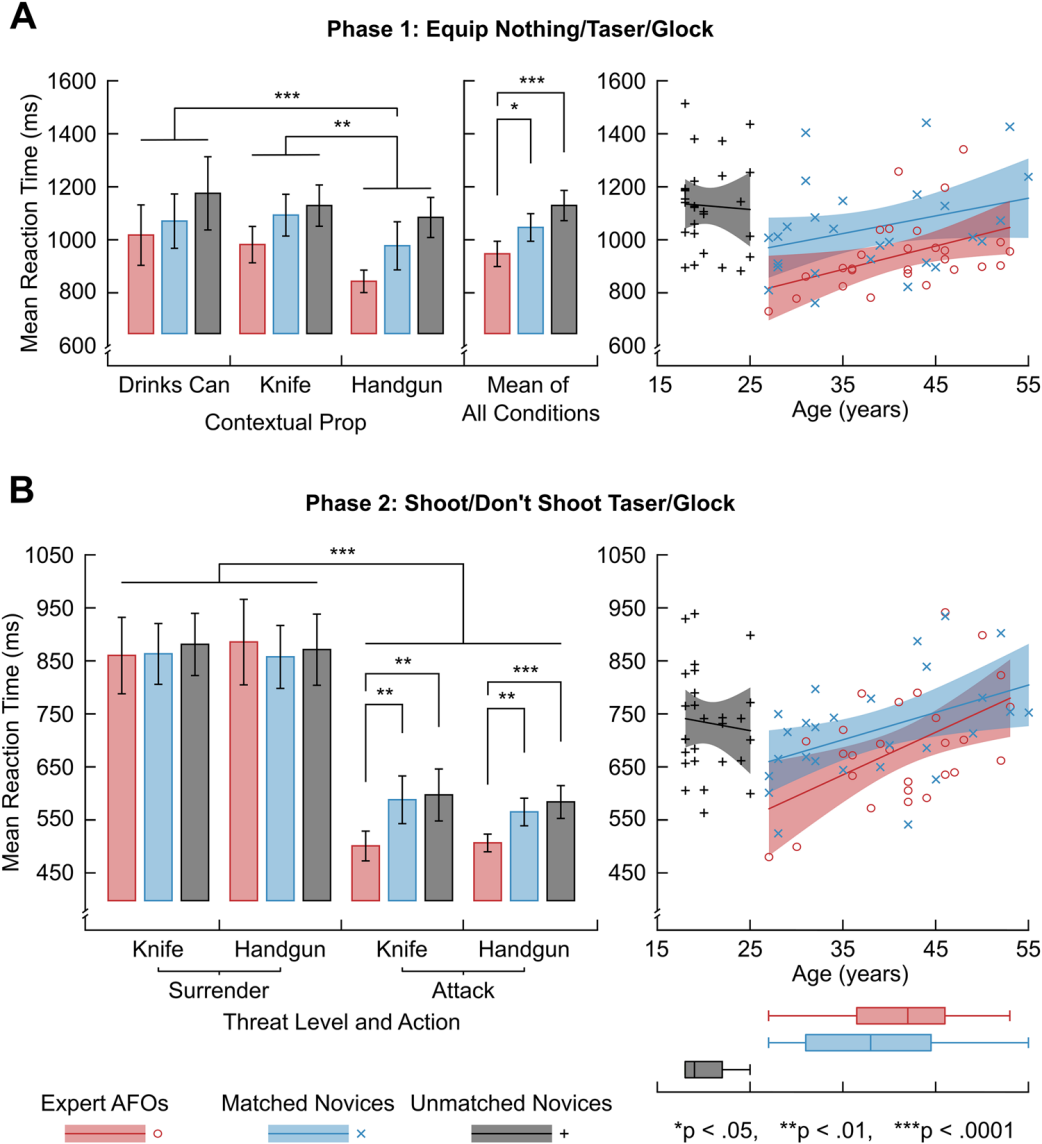
Summary of response time analysis at the two decision points. The top panel (***A***) shows the results from Phase 1 of the task, where participants decided to equip either nothing, a Taser, or a Glock. Participants were faster to respond to threat (knife/handgun) than no threat (drinks can), and expert AFO response times were faster than both novice groups at the preparation decision. The bottom panel (***B***) shows the results from the SDS decision at Phase 2. Again, participants were faster to respond to threat (attack) versus no threat (surrender), and expert AFOs were faster to respond to threat than both novice groups at the SDS decision stage. Additional descriptions of the effects of age can be seen on the right of each panel, along with the age distributions of each group shown with box-and-whisker plots of the interquartile range. Note that the error bars on bar charts show the standard error of the mean and the black bars highlight significant differences with asterisked references to the level of significance.

**Figure 3.**
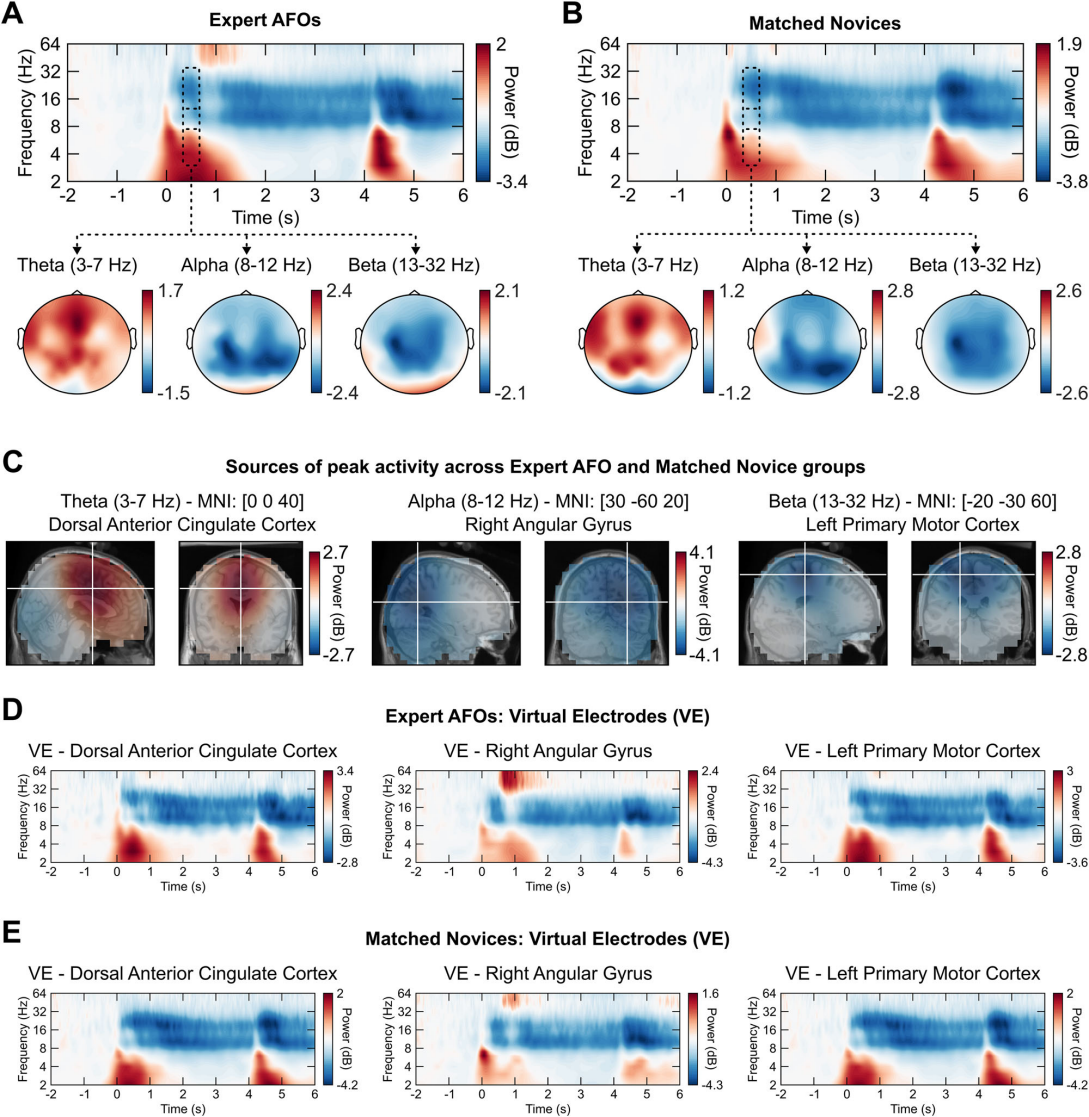
Overview of EEG data preparation and source localization. ***A***, ***B***, The sensor-level time–frequency data for expert AFO and matched novice participants averaged across trials where a weapon was present and the virtual human attacked. For the time–frequency representation figures (top), the average of the central nine electrodes was taken (FC1, FC2, Cz, CP1, CP2, FCz, C1, C2, CPz) and baseline corrected against data from −2 to −1 s using decibel (dB) conversion. In these figures, data are time-locked to the onset of both stimuli, which were always 4 s apart: weapon presence (0 s, Phase 1) and compliance (4 s, Phase 2). On-scalp topographies of frequencies of interest from 250 to 750 ms are shown. ***C***, The source estimation for the theta, alpha, and beta bands using eLORETA. The white crosshairs show the peak activity for each band. ***D***, ***E***, Time–frequency data for virtual electrodes placed at the peaks estimated in ***C***, but for each group separately. Note that all color axes are formed of two linear subscales: from zero to the maximum value and from zero to the minimum value, to highlight the topography of each signal.

**Figure 4.**
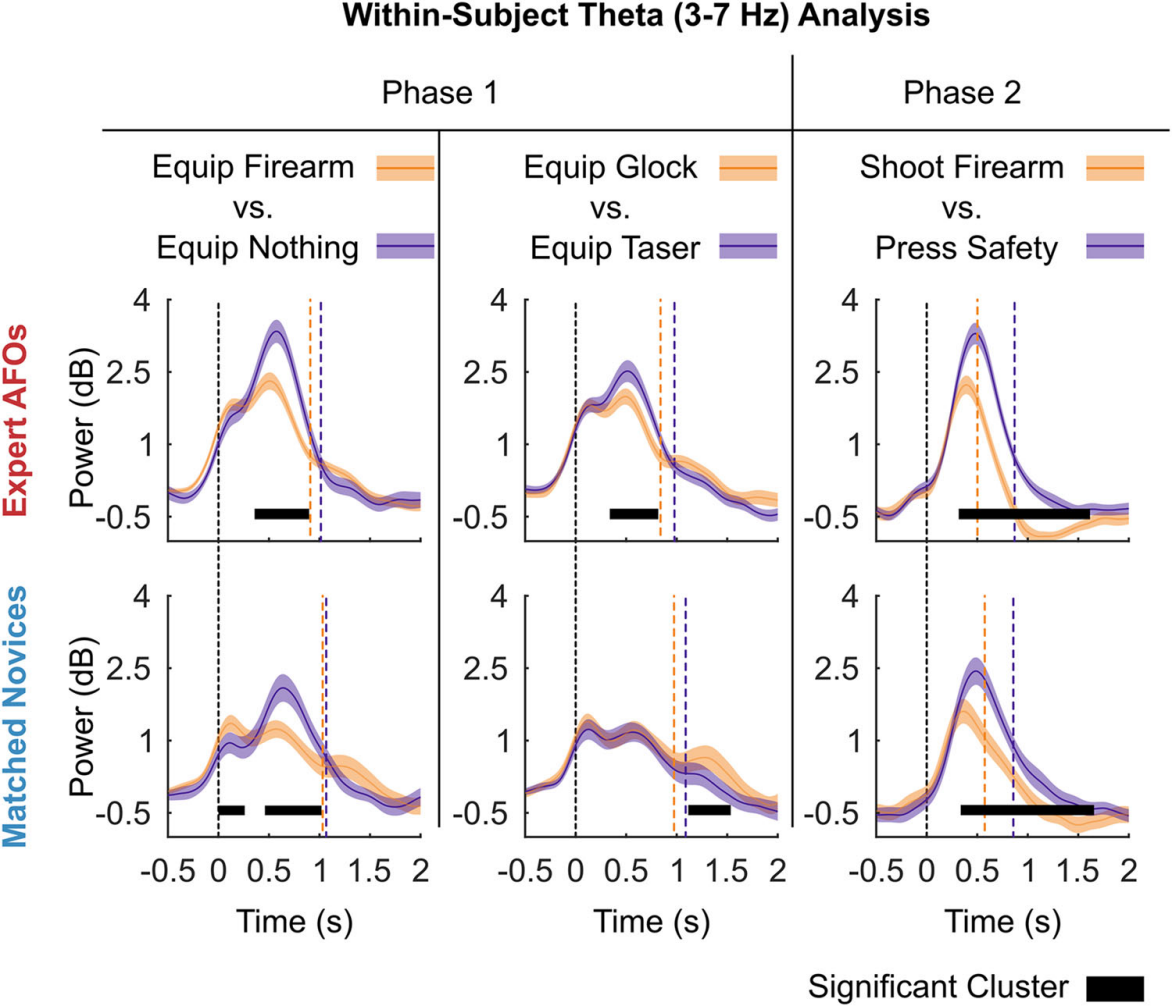
Within-subject comparisons of theta (3–7 Hz) power at a virtual electrode positioned at the positive peak in theta activity averaged across all groups (MNI [0, 0, 40], dorsal anterior cingulate cortex). The right and left panels show that when inhibiting a response (equip nothing, Phase 1, or press safety, Phase 2), both groups of participants exhibit greater dACC theta power versus responding to threat (equip firearm, Phase 1, and shoot firearm, Phase 2). The central panel shows that only expert AFOs demonstrate greater theta power when equipping a Taser versus a Glock at Phase 1. The vertical dashed lines represent stimulus onset (black) and average response times (orange or purple). The black horizontal lines indicate the presence and duration of significant clusters. The shaded areas around the lines show the standard error of the mean.

**Figure 5.**
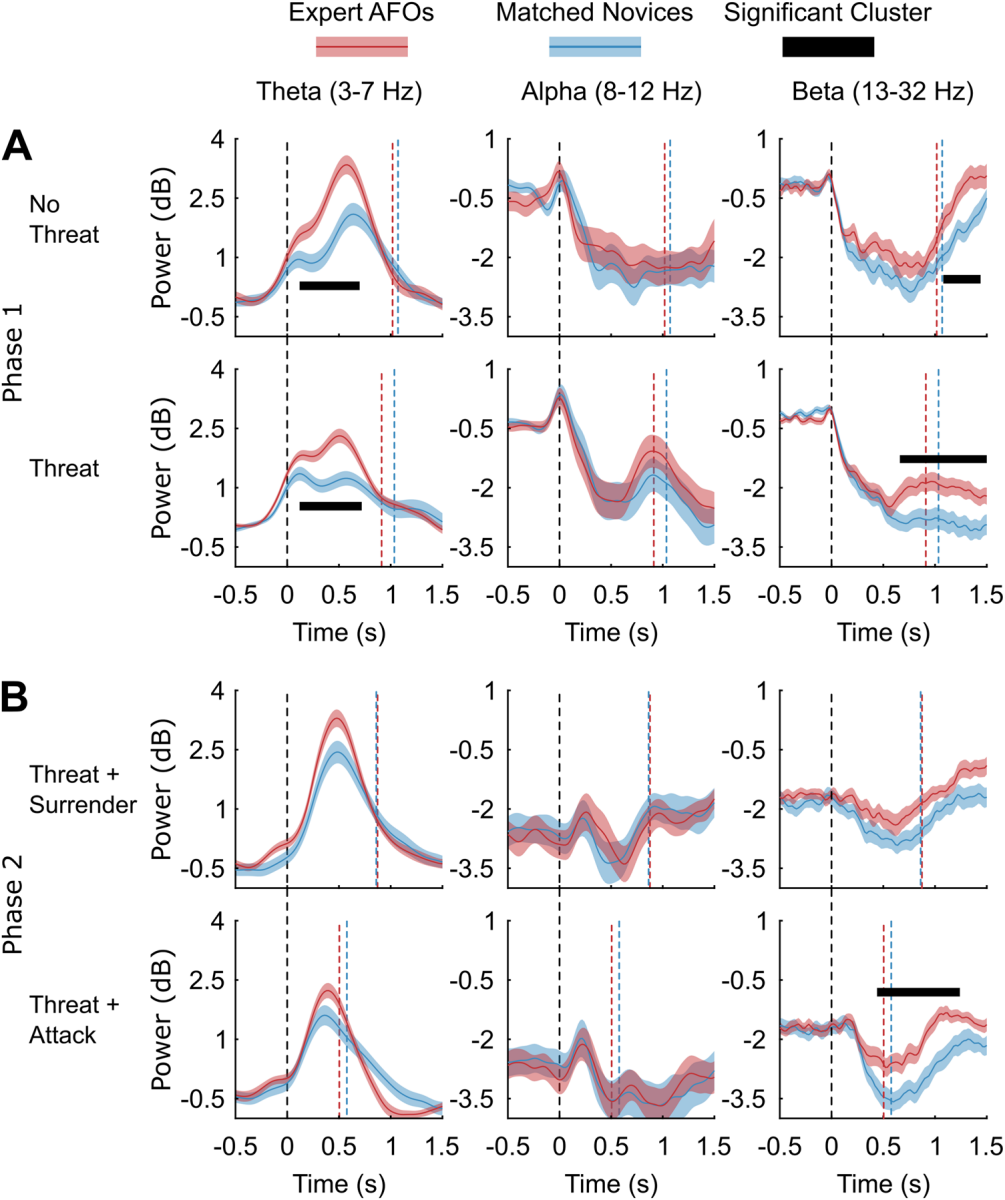
Comparisons of theta, alpha, and beta activity between expert AFO and matched novice groups within critical decision points. In each case, power has been calculated from a virtual electrode placed at the positive (theta) or negative (alpha, beta) peak of oscillatory power in the brain. ***A***, Comparisons from the preparation stage (Phase 1) when there was no threat and when there was a threat. The main finding was that expert AFOs showed significantly higher preresponse theta power than the matched novices in both conditions. A shorter beta rebound was also observed for AFOs compared with matched novices after response in both conditions. ***B***, Comparisons from the SDS decision (Phase 2). No significant preresponse differences were found. A shorter beta rebound was again observed for AFOs compared with matched novices after response, but only in the threat–attack condition. The dashed vertical lines represent stimulus onset (black) and mean group response times (red or blue). The black horizontal lines indicate the presence and duration of significant clusters. The shaded areas around the lines show the standard error of the mean.

We estimated the source in the brain of each frequency band of interest to guide further analysis at the virtual electrode level and to describe the data in the context of associated brain regions. First, baseline (2,000–1,400 ms pretrial) and activity (250–850 ms poststimulus) epochs were defined. The baseline period was selected as the time between trials in which no condition-specific stimulus was presented on screen. Participants were at rest during this period. The activity period was selected based on mean response times across conditions (Fig. 2). The duration of the baseline and activity periods were equal to ensure equal contribution to covariance. A duration of 600 ms was chosen to allow a good estimation of spectral power, even at low frequencies. Power and cross-spectral density within each frequency band were calculated for baseline and activity epochs. These data were then concatenated before calculating the inverse solution using exact low-resolution brain electromagnetic tomography (eLORETA; Pascual-Marqui et al., 1994). The common filter was then applied to the baseline and activity epochs independently before contrasting the two using decibel conversion. This contrast was then averaged across conditions and participants. The coordinates of the maximum power of theta and the minimum power of alpha and beta bands were used in the virtual electrode analysis (Figs. 3*C*, 4, 5).

#### Virtual electrode calculation

To estimate virtual electrode data at the peaks of activity for theta, alpha, and beta, sensor-level data were segmented into epochs from −3,000 to 3,000 ms around the stimulus in conditions of interest. The time-locked average and associated covariance matrix were calculated for each condition. These were used to estimate the inverse solution for the data and the precalculated, individual forward models using a linearly constrained minimum variance (LCMV) beamformer (Van Veen et al., 1997). The inverse solution was applied to the individual epochs to provide virtual electrode data at the previously identified peaks for each condition.

A time–frequency analysis of power was conducted from 1 to 32 Hz (1 Hz resolution) on the virtual electrode data before averaging across trials within each participant and condition (Fig. 3*D,E*). This was done using the “mtmconvol” (multitaper method convolution) frequency analysis in FieldTrip. Despite the name, we used a single Hanning taper for all frequencies. Power at each frequency was calculated within sliding time windows (20 ms resolution). The width of these time windows was set at four times the wavelength of the specified frequency.

Power values were baseline corrected from −2 to −1 s pretrial using decibel (dB) conversion and averaged across frequency, within the frequency bands of interest. Note that baseline correct needed to be applied before statistical analysis of power because variations in impedance between subjects and throughout recordings cause variation in observed power across frequencies. Decibel conversion accounts for this by shifting the power value to a relative measure that is consistent across subjects and over time. Further, in the case of oscillatory power, it shifts the power distribution across time and channels to be normal and within the assumptions of the statistical test used.

### Experimental design and statistical analysis

We used nonparametric cluster-based permutation, as implemented in FieldTrip (Maris and Oostenveld, 2007; Oostenveld et al., 2010), when testing for within- and between-subject comparisons. For within-subject comparisons, a two-tailed dependent sample *t* test was used as the permuted test statistic, and for between-subject comparisons, a two-tailed independent sample *t* test was used. In all cases, 10,000 permutations were taken with a critical alpha level at 0.025 (after two-tailed correction) and cluster alpha level at 0.05 based on the “maxsum” method to correct for multiple comparisons within the test. Note that reported *p*-values are estimates based on the distribution of the permuted cluster statistics. We have reported the temporal bounds of clusters in brackets [start–end], but these should not be considered explicit boundaries. While nonparametric cluster-based permutation tests are suitable to test for differences between data, they may not identify the full extent of a cluster.

### Code accessibility

EEG, behavioral data associated with this study and the code to prepare and analyze them are available for download from the OpenNeuro data-sharing platform (accession number ds004877). They are prepared according to the EEG-BIDS data formatting standard.

## Results

### Behavioral data

#### Matched control groups are essential when comparing expertise

In addition to the Expert AFO and Matched Novice groups, we collected a third dataset of unmatched novices (see Materials and Methods). For each group, we correlated age with response time at Phase 1 and Phase 2 in the SDS task (Fig. 2). We found moderate positive correlations between age and response time for Expert AFOs at Phase 1, *r*_(25)_ = 0.43, *p *= 0.025, and Phase 2, *r*_(25)_ = 0.51, *p *= 0.006. Similar moderate correlations were found for the Matched Novice group at Phase 2, *r*_(25)_ = 0.46, *p *= 0.016, but only negligible correlations were found at Phase 1, *r*_(25)_ = 0.33, *p *= 0.096. Correlations for the Unmatched Novices were also negligible at Phase 1, *r*_(25)_ = −0.05, *p *= 0.82, and Phase 2, *r*_(25)_ = −0.09, *p *= 0.67. These findings are in line with studies of generalized response time and age, which show a plateau in early adulthood, followed by an increasing positive correlation with age (Pierson and Montoye, 1958).

The Expert AFO and Matched Novice groups had only one female participant each, making analysis of the effects of sex inappropriate. The Unmatched Novice group, however, had a more balanced distribution of 12 males and 15 females, allowing for comparisons of the effects of sex of mean response time using unpaired *t*-tests. At Phase 1, no significant difference was found, *t*_(25)_ = 0.744, *p *= 0.46. At Phase 2, males were found to be significantly faster than females, *t*_(25)_ = −2.921, *p *= 0.007. It is important to note that this effect is not applicable beyond the unmatched novice group. We believe these findings highlight that between-subject comparisons of expertise require careful control of participant age and, potentially, sex. This is in addition to established variation in EEG oscillations across age and sex (Hoshi and Shigihara, 2020). Therefore, although we included response time data from the unmatched novice group in the subsequent behavioral analysis, we have focused our analysis of EEG on the expert AFO and matched novice groups.

#### Expert AFOs are quicker to respond at Phase 1

A 3 (between-subject, expertise: Expert AFOs vs Matched Novices vs Unmatched Novices) × 3 (within-subject, weapon presence: Can vs Knife vs Handgun) mixed factor analysis of variance was used to test our hypothesis that response time would differ by expertise and threat. Mauchly's test suggested that the assumption of sphericity was violated for the weapon presence factor, so degrees of freedom were corrected for using Huynh–Feldt estimates of sphericity (*ɛ̃* = 0.78). After sphericity corrections, a significant main effect of weapon presence on response times at Phase 1 was observed, *F*_(1.57,81.55)_ = 9.70, *p *< 0.001, *η*_ges_^2^ = 0.08. A significant main effect of expertise was also found, *F*_(1,52)_ = 5.07, *p *= 0.029, *η*_ges_^2^ = 0.05.

No significant interaction between expertise and weapon presence was found, *F*_(4,156)_ = 0.69, *p *= 0.6, *η*_ges_^2^ = 0.01, and so the direction of the main effects was tested. Pairwise comparisons (after Bonferroni’s correction) between handgun and knife were significant (*p *< 0.001, *d *= 0.49), as were comparisons between handgun and drinks can (*p *= 0.004, *d *= 0.47), but not between knife and drinks can (*p *= 1, *d *= 0.08). This suggests that the main effect of weapon presence was driven by faster response times in the handgun condition only. Similar pairwise comparisons were conducted for the main effect of expertise. Expert AFOs were significantly faster than matched novices (*p *= 0.023, *d *= 0.61). These results show that the observed main effect of group describes AFOs as significantly faster than matched novices.

#### Expertise resulted in faster decisions to shoot at Phase 2

A 3 (between-subject, group: Expert AFOs vs Matched Novices vs Unmatched Novices) × 2 (within-subject, weapon presence: Knife vs Handgun) × 2 (within-subject, action: Surrender vs Attack) mixed factor analysis of variance was conducted. As expected, a main effect of action was found, *F*_(1,78)_ = 315.81, *p *< 0.001, *η*_ges_^2^ = 0.59. The effect size was large, suggesting faster response times for firing in response to being attacked compared with pressing safety in response to a surrender. No significant main effect was found either for weapon presence, *F*_(1,78)_ = 0.31, *p *= 0.58, *η*_ges_^2^ < 0.01, or for group, *F*_(2,78)_ = 1.37, *p *= 0.26, *η*_ges_^2^ = 0.02.

We had expected that Expert AFOs would be faster to respond in the attack condition. Surprisingly, neither a significant main effect for group, *F*_(2,78)_ = 1.37, *p *= 0.26, *η*_ges_^2^ = 0.02 nor a significant interaction between group and action, *F*_(2,78)_ = 2.55, *p *= 0.084, *η*_ges_^2^ = 0.02, were found. However, we conducted an additional pairwise comparison to test for the effect of group on the attack condition of action only as this was not directly tested by the main and interaction effect analyses described. For this test, Levene's test for homogeneity of variance approached significance (*F*_(2,78)_ = 3.09, *p *= 0.051), so we opted to use independent *t* tests without the assumption of equal variance for these pairwise comparisons. This contrast showed that Expert AFOs’ responses to shoot were significantly faster than Matched Novices’, *p *= 0.001, *d *= 1.06.

### EEG data

#### Separation and source localization of oscillations

Comparisons of EEG signals between groups and conditions were made by first identifying and separating signals of interest from the total activity. An overview of this process can be seen in Figure 3. In brief, we estimated the cortical source of three frequency bands of interest, theta, alpha, and beta, independently (see Materials and Methods, Source localization). The coordinates of the maximum power of theta (dorsal anterior cingulate cortex (dACC), MNI [0, 0, 40]) and the minimum power of alpha (right angular gyrus, MNI [30, –60, 20]) and beta (left primary motor cortex, MNI [−20, −30, 60]) versus baseline were then used to generate virtual electrode signals.

#### Theta response is greater in low- versus high-threat conditions

Comparisons between low- and high-threat conditions at Phase 1 and Phase 2 revealed similar differences in theta power at a virtual electrode placed at the estimated peak of activity in the dACC (Fig. 4). At Phase 1, we observed significantly greater preresponse theta when equipping nothing versus equipping a firearm for both the Expert AFO (0.34–0.9 s), *p *< 0.001, and Matched Novice groups (0.44–1.02 s), *p *< 0.001. At Phase 2, we observed significantly greater preresponse theta activity when participants pressed safety versus shooting their firearms for both the Expert AFOs (0.3–1.62 s), *p *< 0.001, and Matched Novices (0.32–1.66 s), *p *< 0.001. In the matched novice group, we also observed an unexpectedly early, significant positive cluster showing greater theta power in the handgun condition (−0.02 to 0.26 s), *p *= 0.039. Given the early timing and near-threshold significance, this is likely a false positive result.

#### Expert AFOs show differences in theta when responding to graded levels of threat

We also contrasted theta activity between trials where participants equipped a Taser versus a Glock to see whether the measure was sensitive to the use of graded levels of force. For the expert AFO group, we found a similar pattern of activity to earlier contrasts which indicated that lower threat level results in greater theta response: expert AFOs had greater preresponse theta activity when equipping a Taser versus a Glock (0.32–0.82 s), *p *= 0.018. For the matched novice group, this contrast did not reveal any significant preresponse clusters. However, we did observe a significant postresponse cluster, showing greater theta power in the Glock condition (1.1–1.54 s), *p *= 0.015.

#### Expert AFOs have greater theta than matched novices when preparing a response to threat

Between-subject comparisons between expert AFOs and matched novices showed that the experts had significantly greater theta activity when responding to threat than novices (0.1–0.72 s), *p *= 0.006. They also had significantly greater preresponse theta when responding to no threat (0.1–0.7 s), *p *= 0.005. See Figure 5*A* for details. Although clusters with the same direction of effect were found when comparing expert AFOs’ and matched novices’ theta activity at Phase 2 (Fig. 5*B*), they were not significant for the surrender (0.3–0.56 s), *p *= 0.065, or attack (0.38–0.48 s), *p *= 0.126, conditions. No significant preresponse differences were found for alpha and beta frequency bands.

#### Expert AFOs show reduced beta desynchronization/faster beta rebound than matched novices

While our hypotheses were focused on comparisons of preresponse theta, analysis of beta band (13–32 Hz) power revealed interesting differences between expert AFOs and matched novices. In both conditions of Phase 1 [no threat (1.06–1.44 s), *p *= 0.015; threat (0.64–1.5 s), *p *= 0.006], experts showed an earlier beta rebound following their response, possibly explained by a smaller initial beta desynchronization preresponse. This effect was replicated at Phase 2 in the threat condition (0.42–1.24 s), *p *= 0.003.

## Discussion

In the current study, expert police AFOs, an age- and sex-matched novice (nonpolice) group, and an unmatched novice group completed an SDS task in VR, in which they had to identify and respond to possible threats in a series of two-phase scenarios. Analysis of response times showed that AFOs consistently performed best, suggesting our task was sensitive to the between-group expertise manipulation. Further, comparisons with the unmatched novice sample included in our study at behavioral level underlined the requirement for matched age distributions in studies comparing control groups with experts (Fig. 2). Subsequent analysis of changes in preresponse oscillatory power at both phases of the SDS task revealed distinct differences between the experts and their matched control group. Most notably, during the preparation phase—when participants determined the appropriate response to varying levels of threat—experts had greater estimated theta power in dACC, suggesting increased orientation toward threatening stimuli.

Our research builds on other studies of police officer decision-making that have used variations of the SDS paradigm (Correll et al., 2002). However, research on EEG signals associated with SDS decision-making has so far been limited, and the patterns of activity are not well understood. Our analysis of EEG data allowed us to first identify the source of signals of interest within the brain and then to measure how the oscillatory activity modulated over time. Generally, our analysis of theta, alpha, and beta bands yielded the expected results across all participant groups (Figs. 4, 5), conforming to demonstrated effects in a variety of traditional experimental paradigms (Pfurtscheller and Lopes da Silva, 1999), as well as recent, naturalistic paradigms (Walshe et al., 2023): When a stimulus is presented to participants and they respond, theta power increases and alpha and beta power decreases.

Having confirmed this expected baseline pattern of activity across groups, we were able to investigate how our experimental design affected these signals. When contrasting EEG-derived virtual electrode signals time-locked to threatening and nonthreatening stimuli, we found that theta power attributed to the dACC was consistently higher across both phases of the experiment and both groups when the stimulus was nonthreatening versus threatening. The estimated source of FMθ activity in the anterior cingulate cortex (ACC) is pertinent to its role in decision-making, as ACC is a well-connected hub of the brain (Cohen, 2011) and part of the executive network (Petersen and Posner, 2012). The ACC is interconnected with the basal ganglia structures of the reward circuit and the ventral striatum (Graybiel and Grafton, 2015; Jin and Costa, 2015; Saga et al., 2017). Through these pathways, faster response times to threat observed in SDS tasks can be attributed to a preferential, adaptive orientation toward threatening stimuli (Lang et al., 1990; Öhman et al., 2001). Modulation of activity at ACC has indeed been observed during SDS tasks and comparable response inhibition paradigms, such as Go/No-Go tasks (Nieuwenhuis et al., 2003; Correll et al., 2006). Our task also shares a common limitation with these tasks in that it is difficult to dissociate performance at the task from motivation. It is possible that observed differences across our expertise manipulation were in fact due to differences in motivation. However, the effect on behavior and associated neural activity is the same. It may be the case that increased motivation and focus are inextricably linked to increasing expertise in these types of tasks.

Subsequent analysis of differences between groups addressed our main research question about the effects of expertise on theta activity attributed to dACC. As expected, AFOs showed greater dACC theta power when assessing the threat in scenarios. This may be related to a more adaptive response to threat, whereby experts are able to reach a decision to respond or inhibit a response quicker than novices. In contrast to our expectations, group differences were not observed for dACC theta nor for alpha or beta frequencies during the second phase of the SDS decision. This may indicate that the initial threat assessment and preparation were crucial in the current scenarios and were significantly influenced by prior training and expertise. Interestingly, however, after SDS responding, AFOs showed shorter beta rebounds compared with matched novices, which could indicate swifter recovery after executing an action (Fig. 5*B*). In other words, AFOs might be “ready for action” after a shorter delay, which could be another reflection of training and expertise.

It is important to note that these findings were obtained within a specific context: Participants were standing and engaging in a task in VR, which required a considerable amount of movement. This required adjustment to the recording protocol and processing of EEG data to minimize and suppress artifacts associated with movement (Klug and Gramann, 2021) and HMD electronics (Weber et al., 2021). Stimulus presentation was naturalistic (Sonkusare et al., 2019), and the timings that our analysis relied on were taken from a continuous presentation of a virtual human in a scene. Further, participants’ interactions with VR affected the stimuli in real time, meaning that how participants chose to respond to the scenario and actions were mapped to realistic movements and so varied between conditions. Therefore, the fact our results are consistent with well-established findings from artificial, highly controlled experiments such as the Go/No-Go task is promising for ongoing research using naturalistic stimuli. Research using artificial stimuli benefits as well: Replication of basic findings in studies using naturalistic stimuli is an important demonstration of the validity of both (Rust and Movshon, 2005). Note that our experimental design was necessarily repetitive and contrived to benefit from a factorial design and sampling of underlying distributions in the data over time. This could certainly have a negative effect on ecological validity. However, by allowing natural behaviors within the bounds of the task, we have shown that realistic behaviors can be observed alongside electrophysiological signals.

Whereas advances in combined VR and neuroimaging (Roberts et al., 2019) and associated analytical methods suggest that many new and interesting research questions can be addressed using naturalistic imaging (De Sanctis et al., 2021), the feasibility of using these methods to address a wide range of research questions varies. Part of the motivation for our research was that, among “natural” expert behaviors, SDS decision-making is particularly amenable to EEG analysis because it occurs in a single, clearly defined moment: the pulling of a trigger or equipping a firearm. This was made clear to us after observing AFO training and observing similarities between their methods and typical studies of human behavior, relating to response time and accuracy. Translating their training into a VR-EEG study enabled us to increase realism to promote ecologically valid behaviors and EEG with a minimal tradeoff for experimental control (Loomis et al., 1999; de la Rosa and Breidt, 2018). Additionally, the use of VR allowed control participants to complete the scenarios without weapon training and outside of specialized training facilities, so novices and experts could participate in the same way. Overall, our study highlights the feasibility of VR-based tasks for investigating police training and expertise more generally. These tasks can be effectively combined with neuroimaging (Tromp et al., 2018), and so we call for a significant increase in “neuro-VR” studies to address the impact of expertise and training on performance.

## Synthesis

Reviewing Editor: James Howard, Brandeis University Decisions are customarily a result of the Reviewing Editor and the peer reviewers coming together and discussing their recommendations until a consensus is reached. When revisions are invited, a fact-based synthesis statement explaining their decision and outlining what is needed to prepare a revision will be listed below. The following reviewer(s) agreed to reveal their identity: Roman Osinsky. Note: If this manuscript was transferred from JNeurosci and a decision was made to accept the manuscript without peer review, a brief statement to this effect will instead be what is listed below.

Accept as is.
